# COPD and its association with smoking in the Mainland China: a cross-sectional analysis of 0.5 million men and women from ten diverse areas

**DOI:** 10.2147/COPD.S75454

**Published:** 2015-03-20

**Authors:** Om P Kurmi, Liming Li, Jenny Wang, Iona Y Millwood, Junshi Chen, Rory Collins, Yu Guo, Zheng Bian, Jiangtao Li, Biyun Chen, Kaixu Xie, Weifan Jia, Yali Gao, Richard Peto, Zhengming Chen

**Affiliations:** 1 Nuffield Department of Population Health, Clinical Trial Service Unit and Epidemiological Studies Unit (CTSU), University of Oxford, Oxford, UK; 2 Department of Epidemiology and Biostatistics, School of Public Health, Peking University, Beijing, Mainland China; 3 Chinese Academy of Medical Sciences, Dong Cheng District, Beijing, Mainland China; 4 China National Center for Food Safety Risk Assessment, Beijing, Mainland China; 5 NCDs Prevention and Control Department, Huixian CDC, Huixian, Henan, Mainland China; 6 NCDs Prevention and Control Department, Hunan CDC, Changsha, Mainland China; 7 NCDs Prevention and Control Department, Tongxiang CDC, Zhejiang, Mainland China; 8 NCDs Prevention and Control Department, Liuyang CDC, Baiyikengdao, Liuyang, Changsha, Hunan, Mainland China; 9 NCDs Prevention and Control Department, Sichuan CDC, Sichuan, Mainland China

**Keywords:** China Kadoorie Biobank, smoking cessation, airflow obstruction, chronic respiratory diseases, Mainland China

## Abstract

**Purpose:**

In adult Chinese men, smoking prevalence is high, but little is known about its association with chronic respiratory disease, which is still poorly diagnosed and managed.

**Methods:**

A nationwide study recruited 0.5 million men and women aged 30–79 years during 2004–2008 from ten geographically diverse areas across the Mainland China. Information was collected from each participant regarding smoking and self-reported physician diagnosis of chronic bronchitis/emphysema (CB/E), along with measurement of lung function indices. Logistic regression was used to yield sex-specific odds ratios (ORs) relating smoking to airflow obstruction (AFO), defined as forced expiratory volume in 1 second (FEV_1_)/forced vital capacity (FVC) <0.7 and CB/E, adjusting for age, areas, education, and income.

**Results:**

Overall 74% of men were ever regular smokers; among them, 7.2% had AFO compared with 5.4% in never-smokers, yielding an OR of 1.42 (95% confidence interval [CI]: 1.34–1.50). The risk was strongly associated with amount smoked and starting to smoke at a younger age. Among ex-smokers, the OR was more extreme for those who had quit due to illness (OR: 1.86, 95% CI: 1.77–1.96) than those who had quit by choice (OR:1.08, 95% CI: 1.01–1.16). CB/E prevalence was also significantly elevated in ex-smokers who had quit because of ill health (OR:2.79, 95% CI: 2.64–2.95), but not in regular smokers (OR:1.04, 95% CI: 0.96–1.11). Female smokers was rare (3%), but carried an excess risk for AFO (OR:1.53, 95% CI: 1.43–1.65) and, to a lesser extent, for CB/E (OR:1.28, 95% CI: 1.15–1.42).

**Conclusion:**

In Mainland China, adult smokers, particularly ex-smokers who had quit because of illness, had significantly higher prevalence of chronic respiratory disease. AFO appeared to be more strongly associated with smoking than self-reported chronic respiratory disease.

## Introduction

Chronic obstructive pulmonary disease (COPD) is characterized by persistent airflow limitation that is usually progressive and often accompanied by a range of comorbidities.^1^ The burden of the disease is particularly high in some low- and middle-income countries, including the Mainland China where it was the third leading cause of years of life lost in 2010.^2^ Despite this high burden of COPD, it is still poorly diagnosed and managed in the Mainland China, particularly in some rural areas.^3^

Tobacco smoking is the primary cause of COPD.^1^ Several large prospective studies have reported that tobacco-attributed mortality is currently much lower in the Mainland China than in Western countries with, for example, relative risk for respiratory mortality in the Mainland China being more than tenfold lower compared to Western countries (<2 versus >20).^4^–^10^ These differences may reflect the older age of initiation and lower smoking intensity in Chinese smokers compared to their Western counterparts,^7^–^9^ and/or potential high background rates of disease in never-smokers.^7^ However, previous prospective studies of smoking in the Mainland China have generally involved relatively small numbers of COPD cases, particularly in women where only a low proportion smoked.^9^,^10^ Moreover, most of these studies in the Mainland China have failed to find any beneficial effect of voluntary smoking cessation on COPD risk.^9^–^13^ This could be attributed to smokers in the Mainland China not quitting until they are critically ill, but no large studies have specifically investigated the associations of recent changes in the amount smoked or the reason for quitting in relation to COPD risk.

Given the known large geographical variation in background rate of COPD across the Mainland China, studies involving multiple and diverse localities will be more informative than those conducted only in a single region. To our knowledge, there has only been one large multicenter study of COPD prevalence in the Mainland China, but that study did not consider associations with smoking in any detail.^13^ We report data from an even larger and more detailed study – the China Kadoorie Biobank (CKB) – that includes spirometric data, self-reported physician-diagnosed chronic bronchitis/emphysema (CB/E), and smoking habits in over 0.5 million men and women from ten diverse regions of the Mainland China.^14^–^17^ The main objectives of the study were 1) to describe cross-sectional associations of tobacco smoking with prevalent spirometrically-defined airway flow obstruction (AFO) in men and women separately; and 2) to investigate the associations of AFO with specific smoking habits, particularly smoking cessation, and in specific strata of the population. In addition, similar analyses were also done for self-reported physician-diagnosed CB/E.

## Methods

### Baseline survey

**Figure 1:**
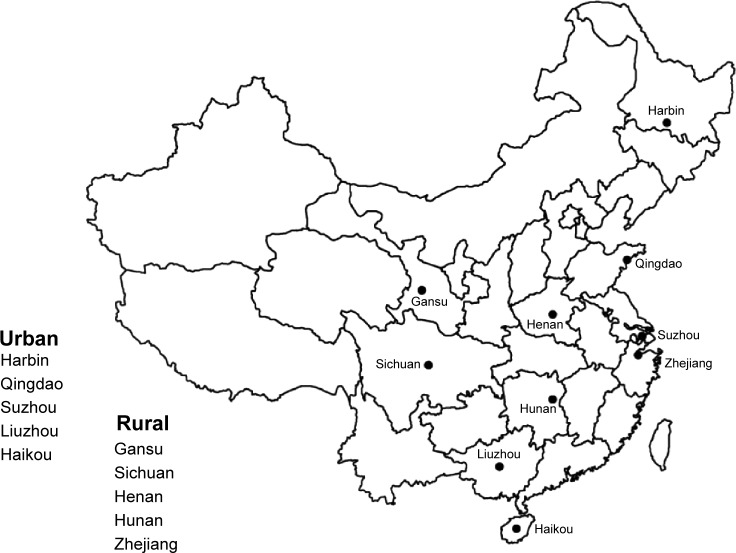
Locations of the ten China Kadoorie Biobank survey sites across Mainland China.

The detailed CKB design and procedures have been described previously.^18^ The baseline survey took place during 2004–2008 in ten geographically defined diverse localities (Figure 1) chosen to include a range of incidence of major chronic diseases (including COPD) and of behavioral and environmental risk factors.^19^ In each area, temporary assessment clinics were set up, and all nondisabled residents aged 35–74 years were invited to participate and ~30% responded. Overall, a total of 512,891 adults were recruited including a few slightly outside the target 35–74 age range group. All participants provided written informed consent and local, national, and international ethical approvals were obtained.

At the study assessment clinics, trained health workers administered laptop-based questionnaires on sociodemographic factors, smoking, alcohol drinking, diet, physical activities, and medical history; measured each participant’s lung function, exhaled carbon monoxide, and blood pressure; and also collected blood for long-term storage.^15^

### Smoking history

Participants were asked about their current and past smoking habits. For the present study, never-smokers were defined as those who had not smoked more than 100 cigarettes during their lifetime. Ever regular smokers were defined as those who had smoked one cigarette or equivalent daily for at least 6 months. Those who had never smoked regularly but had smoked ≥100 cigarettes in their lifetime were classified as occasional smokers. Regular smokers who had quit completely for at least 6 months at baseline were classified as ex-smokers.

For ever regular smokers, additional information was also collected, including age started to smoke regularly, amount and types of tobacco smoked when last smoking, depth of inhalation, and recent changes in amount smoked, and for ex-smokers the main reason for quitting (ill health, concerns about effect on future health, money worries, pressure from family, and other unspecified reasons) was recorded. The amount of tobacco smoked (g/day) by each smoker was calculated, assuming 1 g of tobacco per factory cigarette and 2 g per cigar, with quantities smoked in pipes and hand-rolled cigarettes given as liang/month (1 liang equivalent to 50 g) by the respondents. To help validate smoking exposure, exhaled carbon monoxide (CO) was measured in all participants, using the MicroCO meter (CareFusion Corp, San Diego, CA, USA).^20^

### Spirometry and COPD

Forced expiratory volume in 1 second (FEV_1_) and forced vital capacity (FVC) were measured using a hand held Micro (MS01) Spirometer (CareFusion Corp) by trained technicians following recommended procedures.^21^ Participants made some practice blows, then the results of two successful blows were recorded. Participants were classified to their AFO status using modified Global Initiative for Chronic Obstructive Lung Disease (GOLD) spirometric criteria (prebronchodilator FEV_1_/FVC <0.7).^1^ The presence of AFO includes both asthma and COPD, as no bronchodilator was used, although the self-reported physician-diagnosed prevalence of asthma in the CKB participants was extremely low (~0.5%). During the survey, participants were also asked if they had ever had a diagnosis of CB/E (ie, COPD) by a physician.

Of the 512,891 (210,259 men, 302,632 women) participants enrolled, 396 (202 men, 194 women) with a recorded FEV_1_/FVC >1 were excluded, leaving 210,057 men and 302,438 women for the main analyses. The highest FEV_1_ and FVC values, not necessarily always from the same blow, were used in the analyses.^22^

### Statistical analysis

Given the large difference in smoking prevalence, all analyses were conducted separately for men and women. The prevalence of AFO and CB/E was calculated for the different categories of smoking, directly standardized to the age group structure (30–39 years, 40–44 years, 45–49 years, 50–54 years, 55–59 years, 60–64 years, 65–69 years, and 70–79 years) and region structure of the male or female population.

Logistic regression was used to estimate the odds ratios (OR) of AFO or CB/E associated with smoking, adjusting for the 5-year age group, region, annual household income (<5, 5–9, 10–19, 20–34, ≥35 thousand yuan/year), highest education level (no formal, primary, secondary, tertiary), and the region by age group interaction. For variables with more than two groups, the ORs and their 95% confidence intervals (CI) were floated so that comparisons could be made between any chosen groups rather than just with the baseline category.^23^ Heterogeneity or trends of ORs between different smoking categories were assessed with chi-square tests.

Effect modification was assessed by logistic regression analysis within subgroups of urban/rural region, baseline age, and category of household income. All analyses used SAS 9.3 (SAS Institute Inc., Cary, NC, USA).

## Results

Smoking prevalence differed considerably between men and women. In men, 74.3% were ever regular smokers compared to only 3.2% in women. In both sexes, smokers were less well educated and had a lower household income (Table 1). Compared with female regular smokers, male smokers had, on average, started smoking ~5 years earlier (22.5 years versus 27.1 years, for men and women, respectively), smoked twice as much (18.3 g versus 9.6 g tobacco/day, respectively) and were more likely to inhale to the lung (35.9% versus 25.3%, respectively) (Tables 2 and 3).

**Figure 2:**
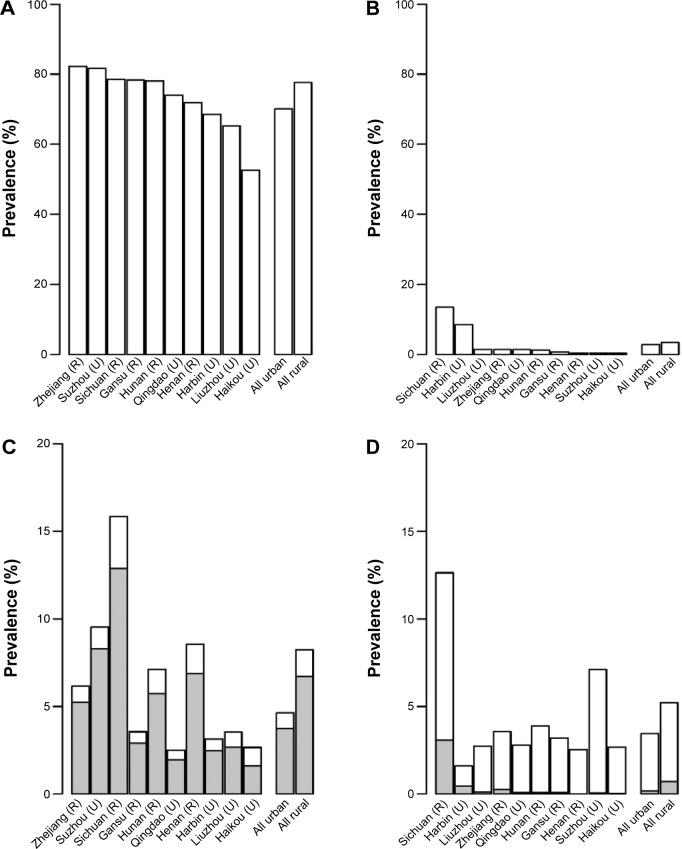
Prevalence of smoking and airflow obstruction in regular smokers in the ten areas. **Notes:** (**A** and **B**) Prevalence of regular smoking in men and women, respectively. (**C** and **D**) Prevalence of airflow obstruction in men and women, respectively. The percentage of each shaded bar represents the percent of airflow obstruction cases in that region for smokers. Bars for all plots are order by the regional prevalence of regular smoking in men or women. **Abbreviations:** R, rural; U, urban.

The overall prevalence of AFO was 6.7% (number [n]=14,024) in men and 4.4% (n=13,387) in women, and varied considerably across ten areas, with the crude prevalence ranging from 2.5%–15.9% in men and 1.4%–13.1% in women (Figure 2). The prevalence of CB/E was about half that of AFO in both men (3.1%; n=6,538) and women (2.2%; n=6,741). Only 15% of the men and 12% of the women with AFO also had a diagnosis of CB/E, whereas 33% of men and 23% of women with CB/E also had AFO. Regular smokers had a higher prevalence of AFO (Tables 2 and 3). In men, 7.2% of regular smokers had AFO versus 5.4% in never-smokers, yielding an adjusted OR of 1.42 (95% CI: 1.34–1.50). Similarly, in women, these were 6.4% versus 4.3%, yielding an adjusted OR of 1.53 (95% CI: 1.43–1.65). In both sexes, the OR was more extreme in those who started to smoke at a younger age (*P*-trend <0.0001 in men and 0.0063 in women). The association with the amount smoked was positive but weak in men and statistically significant in women. CB/E was not associated with regular smoking in men (OR: 1.04; 95% CI: 0.96–1.11), but there was a weak positive association in women (OR: 1.28; 95% CI: 1.15–1.42) (Tables 2 and 3).

At baseline, 17.9% of male regular smokers and 26.9% of female regular smokers were ex-smokers, with about half reporting that they had quit because of ill health. Compared with current smokers, ex-smokers were older in both men (57.8 years versus 51.7 years for ex-smokers and current smokers, respectively) and women (63.4 versus 58.9 years, respectively), and overall ORs of AFO were only slightly elevated in ex-smokers (Tables 2 and 3). However, among those who had quit because of ill health, the ORs were considerably elevated in both men (OR: 1.86; 95% CI: 1.77–1.96) and women (OR: 2.03; 95% CI: 1.78–2.31). Similar findings were also seen for CB/E (men, OR: 2.79, 95% CI: 2.64–2.95; women, OR: 3.24; 95% CI: 2.78–3.78). There was little or no excess risk of either AFO or CB/E among those who had quit by choice, but regular smokers who had recently decreased the amount of smoking had comparable excess risk to those who quit because of ill health: in men the OR for AFO was 1.77 (95% CI: 1.68–1.87) and in women it was 1.90 (95% CI: 1.61–2.24). For CB/E, similar findings were also evident, though to a lesser extent (Tables 2 and 3).

Although AFO prevalence in never-smokers was lower in urban than in rural areas, the association of AFO with regular smoking was more extreme in urban areas for both men and women. The OR of AFO also increased with increasing age, probably mainly reflecting the effects of increased smoking duration (Table 4). AFO prevalence was inversely associated with annual income in never-smokers, but the OR of AFO for regular versus never-smokers was more extreme in those with higher household income (Table 4).

## Discussion

In this large nationwide study of smoking and chronic respiratory disease among adult men and women in the Mainland China, the prevalence of spirometrically-defined AFO, as well as self-reported CB/E among never-smokers varied considerably across the ten areas of study. Compared with never-smokers, male regular smokers had, on average, about 50% excess risk of AFO and the disease prevalence was associated strongly and positively with the amount smoked and a younger age of starting to smoke regularly. For AFO, the OR was particularly large among smokers who had quit due to illness or those who had recently reduced the amount smoked. Among women, despite an extremely low smoking prevalence, smokers also had significant excess risk of AFO and CB/E.

Although our study comprised of cross-sectional analyses of baseline data from a prospective study, the observed excess risk of AFO among regular smokers was broadly consistent with the results of previous large prospective studies of smoking and cause-specific mortality in the Mainland China, and with the two meta-analyses of Asian prospective studies,^6^–^10^ which showed a 50% excess respiratory mortality in smokers and elevated risks in ex-smokers similar to those in current smokers.^9^,^10^ Our results are also in agreement with the findings from two other much smaller cross-sectional studies from the Mainland China on smoking with physician-diagnosed COPD or spirometrically-defined COPD.^13^,^24^

The large number of prevalent cases in our study allowed for the appropriate assessment of association between prevalent COPD cases with smoking status, frequency and habits, not only overall, but also in certain subgroups of the study population. The risk associated with smoking varied by area, age, sex, and income. The increasing trend of OR with increasing baseline age probably mainly reflected the effects of a longer duration of smoking among older smokers. Compared with younger smokers, however, the older smokers were less likely to start to smoke regularly at an earlier age, less likely to ever smoke cigarettes, less likely to smoke a large number of cigarettes/day, and less likely to inhale to the lung, all limiting their risk. The tobacco-attributed risk is more likely to increase significantly in the Mainland China when the older generation is replaced by younger ones. Similarly, the stronger associations of AFO with smoking in urban areas and in those with higher annual household incomes probably also reflected, at least in part, differences in past smoking patterns. The excess risk of AFO associated with smoking was similar in women compared to men, despite the fact that female smokers had started at an older age and smoked only about half as much – compared to their male counterparts. Other studies, including a Chinese study, have also reported similar findings.^24^,^25^ Further investigations of this apparent greater susceptibility of female smokers to COPD are needed.

To our knowledge, the present study is the only large study of AFO and smoking that contained detailed information on smoking cessation and recent changes in the amount smoked. Our results are in line with several earlier small studies of Chinese and Asian smokers that have found elevated risks of COPD (but not other diseases) in ex-smokers compared to current smokers.^9^,^10^,^12^,^13^ The significant excess risk of AFO among smokers who had quit because of ill health in the present study confirms the suggested notion that many smokers do not attempt to quit until they are ill with COPD. This is further supported by the elevated risk of AFO observed among smokers who had recently reduced their smoking intensity. In contrast, the little excess risk among ex-smokers who had quit by choice rather than due to ill health is consistent with the beneficial effect of quitting smoking that has been observed in Western populations.^11^

In our study, CB/E was associated only weakly with regular smoking, but strongly with quitting smoking because of ill health. Less than 15% of participants with AFO also had CB/E, suggesting that there may be considerable underdiagnosis of this condition in the population. In contrast to AFO, the general lack of association with regular smoking suggests an even greater degree of underdiagnosis of CB/E in regular smokers than in never-smokers. It is possible that smokers may be relatively unaware or unwilling to accept the serious health implications of early respiratory symptoms.

As well as several important strengths, our study also has a number of limitations. First, the association is based on cross-sectional data so we cannot be sure about the direction of causality, but smoking has been established as the main risk factor for COPD.^4^,^5^,^15^ In CKB, the mean age of starting to smoke was 22 years in men and 27 years in women, compared to a mean age of >40 years for the initial diagnosis of CB/E in both sexes. Therefore, reverse causality is probably not a concern in comparing regular smokers to never-smokers. However, the possibility of reverse causality or recall bias in the reporting of specific smoking habits by regular smokers cannot be excluded, in particular in the amount smoked at baseline. A tendency to adjust tobacco consumption in response to respiratory symptoms might also explain the relatively weak association of AFO with daily tobacco consumption at baseline. Second, as we did not use a bronchodilator, we may have overestimated the prevalence of COPD, but the degree of overestimation is likely to be small as the prevalence of asthma is quite low in the Mainland China when compared to Western countries.^26^,^27^ In addition, bronchodilator reversibility does not always distinguish well between asthma and COPD.^28^ Third, the spirometer used at the baseline survey did not produce a spirogram to allow for the assessment of the acceptability of blows, even though participants were carefully instructed and requested to make practice blows during the survey. Incomplete inhalation or early termination of a blow would have resulted in a reduced FVC and hence in the underestimation of AFO. If this misclassification were nondifferential across categories of smokers, we would expect it to bias associations towards the null.

With rapid economic development and improvements in living standards in the Mainland China, the age-standardized COPD rates in never-smokers may well decrease significantly over time. However, the present study showed that although the proportional risk of COPD associated with smoking is currently still very modest, the overall burden of tobacco-attributed COPD is already high because of the high background rate in the population. The prevalence of smoking in women is very low and is decreasing, but the relative risk of COPD in female smokers appears at least as great as in male smokers. Quitting smoking could have a beneficial effect on COPD providing it is done before ill health develops, but our data suggest that many smokers in the Mainland China did not fully appreciate the risks of smoking and tended to quit only after they had developed serious illness.

## Figures and Tables

**Table 1:** Baseline characteristics of the study participants by smoking category

Variable*	Men (n=210,057)			Women (n=302,438)		
	Never	Occasional	All regular	Never	Occasional	All regular
All (N)	30,282	23,610	156,165	287,117	5,522	9,799
Study area						
Urban	55.5	44.4	40.9	44.8	38.6	39.4
Rural	44.5	55.6	59.1	55.2	61.4	60.6
Age at baseline (years)	54.4±11.9	51.1±11.1	52.8±10.6	51.1±10.4	53.1±10.9	60.1±9.9
30–39	15.2	19.9	13.0	16.4	13.3	3.6
40–49	23.2	29.7	28.9	31.5	28.3	13.4
50–59	25.8	26.9	31.7	31.2	29.3	27.3
60–69	23.8	16.8	19.3	15.8	21.3	38.5
70–79	12.1	6.7	7.1	5.1	7.8	17.2
Annual household income (yuan; in thousands)						
<5	8.4	8.6	9.5	9.4	20.2	25.6
5–9	14.9	16.6	17.2	19.4	23.4	25.0
10–19	28.4	28.9	28.2	29.6	29.6	28.8
20–34	26.7	24.6	25.3	24.7	16.8	13.4
≥35	21.6	21.2	19.8	16.9	10.1	7.3
Highest education completed						
No formal school	7.3	5.8	9.6	25.0	21.2	34.9
Primary school	26.6	28.0	35.5	31.0	35.3	41.1
Middle/high school	50.0	54.4	49.2	39.4	38.6	22.6
College/university	16.1	11.8	5.7	4.5	4.9	1.4
Standing height (m)	1.65±0.07	1.65±0.06	1.65±0.06	1.54±0.06	1.53±0.07	1.52±0.06
FEV_1_ (L)	2.61±0.68	2.77±0.66	2.65±0.70	2.00±0.48	1.92±0.57	1.64±0.54
FVC (L)	3.06±0.75	3.27±0.74	3.16±0.77	2.36±0.54	2.31±0.60	2.03±0.57
FEV_1_/FVC (%)	85.5±8.9	84.6±8.1	83.6±9.3	85.0±7.8	82.7±9.1	80.2±10.2

**Note:**

* Data are % or mean ± SD.

**Abbreviations:** n, number; N, total number; FEV_1_, forced expiratory volume in 1 second; FVC, forced vital capacity; SD, standard deviation.

**Table 2:** Associations of smoking with prevalent AFO and self-reported chronic bronchitis/emphysema among men

	Smoking habit (%)	AFO		Bronchitis/emphysema	
		%‡	OR (95% CI)†	%‡	OR (95% CI)†
**Smoking category**					
Never	14.4	5.4	1.00 (0.95–1.06)	3.3	1.00 (0.94–1.07)
Occasional	11.2	5.2	0.93 (0.88–1.00)	3.1	0.98 (0.90–1.06)
Exregular	13.3	7.4	1.50 (1.44–1.57)	5.6	1.85 (1.77–1.95)
Current regular	61.1	7.1	1.37 (1.34–1.41)	2.5	0.77 (0.74–0.80)
*P* heterogeneity			<0.0001		<0.0001
All regular	74.4	7.2	1.42 (1.34–1.50)	3.1	1.04 (0.96–1.11)
**Amount smoked (g tobacco/day) in regular smokers**					
Mean (SD), 18.3 (10.8)					
<5	6.1	6.6	1.34 (1.24–1.44)	3.3	0.99 (0.87–1.11)
5–14	28.9	6.9	1.36 (1.31–1.41)	2.9	0.95 (0.90–1.01)
15–24	46.2	7.4	1.46 (1.42–1.51)	3.2	1.03 (0.99–1.08)
25–34	10.4	7.6	1.47 (1.39–1.56)	3.4	1.11 (1.02–1.21)
≥35	8.4	7.4	1.42 (1.32–1.52)	4.3	1.32 (1.21–1.45)
*P* trend			0.014		<0.0001
**Age started to smoke regularly (years) in regular smokers**					
Mean (SD), 22.5 (7.0)					
≥28	6.3	5.9	1.15 (1.10–1.21)	4.2	0.76 (0.70–0.81)
24–27	27.7	6.5	1.27 (1.21–1.34)	3.7	0.92 (0.85–0.99)
20–23	32.9	7.4	1.48 (1.43–1.54)	3.4	1.12 (1.07–1.18)
16–19	16.1	8.1	1.60 (1.54–1.67)	2.8	1.24 (1.17–1.31)
<16	16.9	9.6	1.79 (1.68–1.92)	2.4	1.35 (1.22–1.49)
*P* trend			<0.0001		<0.0001
**Depth of inhalation in regular smokers**					
Mouth/throat	51.8	7.0	1.35 (1.31–1.39)	2.9	0.94 (0.90–0.98)
Lung now	12.3	6.4	1.27 (1.19–1.35)	3.2	1.06 (0.97–1.15)
Lung always	35.9	7.7	1.58 (1.52–1.64)	3.6	1.19 (1.13–1.25)
*P* trend			<0.0001		<0.0001
**Recent changes in amount smoked in current regular smokers**§					
No change	66.5	6.8	1.28 (1.24–1.32)	2.1	0.62 (0.59–0.65)
Increased a lot	15.3	6.9	1.33 (1.25–1.42)	2.4	0.72 (0.64–0.80)
Decreased a lot	12.7	9.0	1.77 (1.68–1.87)	4.3	1.20 (1.10–1.30)
*P* heterogeneity			<0.0001		<0.0001
**Reason for stopping smoking in exregular smokers**					
Ill health	49.6	8.8	1.86 (1.77–1.96)	8.4	2.79 (2.64–2.95)
Other reason	50.4	5.6	1.08 (1.01–1.16)	2.7	0.90 (0.81–0.99)
*P* heterogeneity			<0.0001		<0.0001

**Notes:**

† Adjusted for region, 5-year age group, age group × region interaction, education, and income, with the never-smoker category as the reference group. With the exception of the comparison of all regular versus never-smokers, the CIs are floated, so that comparisons can be made between any pair of categories.

‡ Prevalences of disease are directly standardized to the age and area structure of the study’s male population.

§ The variable was missing in 5.4% of male current smokers (N=6,963 men).

**Abbreviations:** AFO, airflow obstruction; OR, odds ratio; CI, confidence interval; SD, standard deviation; N, total number.

**Table 3:** Associations of smoking with prevalent AFO and self-reported chronic bronchitis/emphysema among women

	Smoking habit (%)	AFO		Bronchitis/emphysema	
		%‡	OR (95% CI)†	%‡	OR (95% CI)†
**Smoking category**					
Never	94.9	4.3	1.00 (0.96–1.04)	2.2	1.00 (0.95–1.05)
Occasional	1.8	4.4	1.11 (1.00–1.22)	1.9	0.99 (0.84–1.15)
Exregular	0.9	5.0	1.67 (1.50–1.86)	4.0	2.31 (2.02–2.63)
Current regular	2.4	6.5	1.46 (1.36–1.57)	2.0	0.87 (0.77–0.99)
*P* heterogeneity			<0.0001		<0.0001
All regular	3.3	6.4	1.53 (1.43–1.65)	2.4	1.28 (1.15–1.42)
**Amount smoked (g tobacco/day) in regular smokers**					
Mean (SD), 9.6 (7.5)					
<5	27.4	6.3	1.38 (1.23–1.55)	2.2	1.14 (0.96–1.36)
5–14	48.9	5.6	1.64 (1.51–1.79)	2.6	1.32 (1.16–1.50)
15–24	20.5	7.1	1.48 (1.30–1.69)	2.3	1.37 (1.13–1.67)
25–34	2.0	9.2	1.58 (1.07–2.34)	2.3	1.49 (0.84–2.63)
≥35	1.2	9.8	1.79 (1.03–3.10)	5.9	1.10 (0.44–2.70)
*P* trend			0.269		0.1966
**Age started to smoke regularly (years) in regular smokers**					
Mean (SD), 27.1 (11.9)					
≥28	11.3	5.5	1.36 (1.23–1.52)	1.4	1.00 (0.85–1.18)
24–27	18.5	6.7	1.57 (1.33–1.86)	2.5	1.27 (0.98–1.66)
20–23	16.8	7.6	1.69 (1.47–1.93)	3.8	1.56 (1.28–1.90)
16–19	12.2	7.9	1.50 (1.32–1.70)	3.1	1.52 (1.26–1.84)
<16	41.1	9.4	1.82 (1.56–2.11)	1.9	1.45 (1.15–1.84)
*P* trend			0.0063		0.0005
**Depth of inhalation in regular smokers**					
Mouth/throat	62.0	6.4	1.49 (1.39–1.61)	2.2	1.14 (1.02–1.29)
Lung now	12.6	7.0	1.68 (1.41–1.99)	2.2	1.28 (0.99–1.66)
Lung always	25.3	6.9	1.58 (1.39–1.78)	3.4	1.65 (1.40–1.95)
*P* trend			0.3214		0.0004
**Recent changes in amount smoked in current regular smokers**§					
No change	62.5	6.3	1.37 (1.25–1.51)	1.8	0.69 (0.58–0.83)
Increased a lot	16.7	8.2	1.55 (1.30–1.86)	3.4	1.04 (0.77–1.41)
Decreased a lot	14.1	11.0	1.90 (1.61–2.24)	2.9	1.26 (0.96–1.65)
*P* heterogeneity			0.0056		0.0003
**Reason for stopping smoking in ex regular smokers**					
Ill health	54.6	6.5	2.03 (1.78–2.31)	9.3	3.24 (2.78–3.78)
Other reason	45.4	5.2	1.20 (0.99–1.45)	2.0	1.22 (0.95–1.58)
*P* heterogeneity			<0.0001		<0.0001

**Notes:**

† Adjusted for region, 5-year age group, age group × region interaction, education, and income, with the never-smoker category as the reference group. With the exception of the comparison of all regular versus never-smokers, CIs are floated, so that comparisons can be made between any pair of categories.

‡ Prevalences of disease are directly standardized to the age and area structure of the study female population.

§ The variable was missing in 6.8% of female current smokers (N=485 women).

**Abbreviations:** AFO, airflow obstruction; OR, odds ratio; CI, confidence interval; SD, standard deviation; N, total number.

**Table 4:** ORs for prevalent AFO and self-reported chronic bronchitis/emphysema in regular versus never-smokers by sociodemographic factors

	Characteristics of regular smokers (crude mean or prevalence)					AFO			Bronchitis/emphysema		
	Prevalence (%)	Age started (years)	Age at baseline (years)	Amount smoked (g tobacco/day)	Always inhaled to lungs (%)	Prevalence (%)‡		OR (95% CI)†	Prevalence (%)‡		OR (95% CI)†
						Never-smokers	Regular smokers		Never-smokers	Regular smokers	
**Men**											
Study area											
Urban	70.1	22.6	52.9	18.0	42.7	3.1	5.2	1.72 (1.56–1.90)	3.2	3.1	1.13 (1.01–1.26)
Rural	77.6	22.4	52.8	18.6	31.1	7.0	8.7	1.28 (1.19–1.37)	3.3	3.2	0.96 (0.87–1.06)
*P* trend								<0.0001			0.0311
Age at baseline, years											
<50	73.7	20.9	42.6	18.7	38.1	2.2	2.5	1.11 (0.96–1.27)	1.8	1.2	0.73 (0.62–0.87)
50–59	77.8	22.7	54.7	19.0	36.4	4.9	6.3	1.30 (1.15–1.46)	3.3	2.6	0.82 (0.71–0.95)
60–69	73.0	24.6	64.7	17.3	31.8	9.1	13.2	1.51 (1.37–1.66)	5.1	5.8	1.17 (1.03–1.32)
70–79	68.0	25.1	72.5	15.6	31.3	14.9	21.2	1.63 (1.45–1.83)	6.5	8.1	1.39 (1.18–1.62)
*P* trend								<0.0001			<0.0001
Annual household income (yuan, thousands)											
<5	76.5	23.3	58.7	17.0	27.7	9.3	9.5	1.33 (1.17–1.52)	5.1	3.7	1.08 (0.88–1.32)
5–9	76.0	22.7	52.9	17.7	34.8	6.6	8.3	1.26 (1.11–1.44)	3.7	3.4	0.91 (0.76–1.08)
10–19	74.1	22.2	52.7	18.3	38.1	6.1	7.3	1.34 (1.20–1.49)	3.2	3.0	1.07 (0.93–1.24)
20–34	74.0	22.2	51.8	18.6	37.2	4.1	6.1	1.62 (1.41–1.85)	3.2	3.2	1.02 (0.87–1.18)
≥35	72.9	22.7	51.3	19.1	35.8	3.3	5.5	1.81 (1.53–2.14)	4.5	2.8	1.10 (0.93–1.30)
*P* trend								0.0005			0.5173
**Women**											
Study area											
Urban	2.9	29.5	61.0	9.7	29.1	3.4	5.5	2.04 (1.75–2.37)	2.4	2.6	1.51 (1.28–1.78)
Rural	3.5	25.6	59.6	9.5	22.9	5.0	7.1	1.44 (1.33–1.56)	2.0	2.3	1.17 (1.02–1.34)
*P* trend								<0.0001			0.0195
Age at baseline, years											
<50	1.2	27.5	43.9	10.1	31.0	2.3	2.9	1.44 (1.14–1.81)	1.3	0.9	0.88 (0.57–1.36)
50–59	2.9	26.3	55.0	10.0	25.8	4.0	6.7	1.35 (1.18–1.54)	2.4	2.8	1.10 (0.88–1.37)
60–69	7.5	27.3	65.4	9.4	23.4	7.5	12.0	1.74 (1.56–1.94)	3.6	4.9	1.45 (1.25–1.69)
70–79	10.1	27.7	72.5	9.0	23.3	13.4	18.8	1.61 (1.38–1.87)	4.6	7.6	1.47 (1.17–1.84)
*P* trend								0.0497			0.0099
Annual household income (yuan, thousands)											
<5	8.2	25.4	62.4	9.6	20.3	5.8	7.2	1.38 (1.22–1.55)	2.8	1.8	1.10 (0.90–1.33)
5–9	4.1	26.5	58.9	9.7	28.0	4.8	6.4	1.38 (1.21–1.59)	2.3	2.5	1.26 (1.02–1.57)
10–19	3.2	27.2	59.5	9.7	26.6	4.1	6.0	1.72 (1.49–1.98)	2.2	2.5	1.28 (1.04–1.57)
20–34	1.8	30.0	60.1	9.5	28.0	3.6	6.2	1.95 (1.57–2.43)	2.3	3.6	1.73 (1.32–2.27)
≥35	1.4	29.7	59.3	9.5	24.2	3.5	7.6	1.90 (1.40–2.59)	2.4	1.5	1.22 (0.82–1.84)
*P* trend								0.0005			0.0553

**Notes:**

† ORs are for all regular smokers versus never-smokers, adjusted for 5-year age group, region, age group × region interaction, education, and income.

‡ Prevalences of the disease are directly standardized to the age and area structure of the population in the China Kadoorie Biobank.

**Abbreviations:** OR, odds ratio; AFO, airflow obstruction; CI, confidence interval.
